# Alzheimer disease effects of different stages on intestinal flora: A protocol for systematic review and meta-analysis

**DOI:** 10.1097/MD.0000000000028462

**Published:** 2021-12-30

**Authors:** Xunshu Cheng, Haorui Wang, Zhihuang Zheng, Ke Feng, Saixue Tang, Yuanyuan Liu, Ke Chen, Chenhao Bi, Mingzhou Gao, Lijin Ji

**Affiliations:** a College of Traditional Chinese Medicine, Shandong University of Traditional Chinese Medicine, Jinan, Shandong Province, China; b College of Integrative Medicine, Fujian University of Traditional Chinese Medicine, Fuzhou, Fujian Province, China; c College of Traditional Chinese Medicine, Fujian University of Traditional Chinese Medicine, Fuzhou, Fujian Province, China; d Innovative Institute of Chinese Medicine and Pharmacy, Shandong University of Traditional Chinese Medicine, Jinan, Shandong Province, China.

**Keywords:** Alzheimer disease, dementia, gastrointestinal microbiome, META, probiotics

## Abstract

**Background:**

Alzheimer disease (AD) is a common degenerative disease of the central nervous system that can be divided into 3 stages, according to the degree of cognitive impairment. The clinical manifestations are cognitive dysfunction and memory loss, impacting the daily activities of the affected individuals. In recent years, studies have demonstrated a relationship between intestinal flora and AD. However, no meta-analysis has documented the correlation between AD and intestinal flora, to the best of our knowledge. Herein, we sought to assess the correlation between different stages of AD and intestinal flora. A systematic and comprehensive understanding of this relationship is of great significance for developing prevention and treatment strategies against AD.

**Methods:**

A comprehensive search of the medical literature in Chinese and English language was performed in databases, such as PubMed, EBSCO, CNKI, web of science, WanFang, Cochrane Library, and CBM databases. Pre-defined search strategies were used to retrieve clinical studies of Alzheimer disease and gut microbiota. The included studies were independently analyzed by the 2 researchers who extracted the data. The quality of the data was evaluated according to the “Cochrane system evaluator manual.” Finally, Endnote and RevMan software were used for systematic regression and meta-analysis of evidence.

**Results:**

We documented the intestinal flora changes in the 3 stages of Alzheimer disease, according to currently available clinical evidence, and revealed the correlation between the abundance and diversity of flora and treatment efficacy. These findings are essential for developing new strategies for the prevention and treatment of Alzheimer disease.

**INPLASY registration number:**

INPLASY2021100093

**Ethics and dissemination:**

Since all data utilized in this systematic review and meta-analysis are published, ethical approval was not needed.

## 1 Introduction

Alzheimer disease (AD) is a complex senile degenerative disease of the central nervous system and is the predominant type of dementia. Its main clinical manifestations include progressive cognitive dysfunction, memory loss, and amnesia. There are currently about 47.5 million AD patients worldwide, and it is expected to reach nearly 82 million in 2030 and surpass 152 million in 2050.^[1]^ The etiology of AD is still unknown, and its cardinal feature-cognitive dysfunction brings a heavy burden to patients, families, and society.^[2]^ In recent years, AD has become a major public health problem that seriously affects the health and quality of life of the global population.

AD can be divided into 3 stages according to National Institute on Aging-Alzheimer Association (NIA-AA) diagnostic criteria (2011): the preclinical or presymptom stage where patients exhibit mild memory loss and early hippocampal lesions, lasting for several years.^[3]^ The mild cognitive impairment (MCI) stage, where the patient exhibits attention and memory loss, mood changes, depression and anxiety, and other negative emotions that become more serious with cognitive decline.^[4]^ The dementia stage (associated with cerebral cortex lesions), where patients exhibit behavioral (swallowing difficulties and dysuria) and cognitive (unable to identify family members) impairments, and other serious complications.^[5]^ The typical histopathological feature of AD is the accumulation of abnormal protein aggregates, including amyloid plaques consisting of beta-amyloid peptide (Aβ) and neurofibrillary tangles formed by hyperphosphorylated tau proteins.^[6]^ The brain magnetic resonance imaging (MRI) of AD patients is characterized by atrophy of the temporal lobe system, hippocampal volume reduction, enlargement of cerebral sulcus and stenosis of the cerebral gyrus.^[7]^

The gut microbiota forms an important line of defense against GI pathogens and toxins in the human body. It is widely acknowledged that the dynamic balance of gut microbiota plays an important physiological and pathological role.^[8]^ In recent years, the microbiota–gut–brain axis has become the focus of biomedical research on potential therapeutic targets for treating central nervous system diseases,[^9^,^10^] linking the gut microbiota with the brain central nervous system (CNS) through nerve, endocrine, immune, and metabolic pathways, essential for maintaining brain homeostasis.^[11]^ Microbial communities can produce a variety of immunoregulatory substances, which can act on intestinal secretory cells locally and regulate the function of the central nervous system. Brain signals can change the composition of microbial communities and gastrointestinal function via efferent nerves (vagus nerve) and the HPA axis.^[12]^ Emerging studies have shown that metabolites produced by gut microbiota can regulate the differentiation, maturation, and activation of microglia and astrocytes, which mediate a variety of neurophysiological processes, including neurodevelopment, neurotransmission, and CNS immune activation.[^13^,^14^] Mediators secreted by intestinal endocrine cells (EEC) stimulate the vagus nerve to transmit information directly to the brain in response to various mechanical, chemical, and hormonal stimuli from the gut microbiota.^[15]^ Intestinal flora imbalance can increase levels of harmful substances (such as amyloid protein and trimethylamine N-oxide) by enhancing the permeability of the intestinal mucosal barrier and blood-brain barrier, activating the peripheral immune response and causing amyloid plaque formation to promote the pathological progress of AD.^[16]^

Probiotics are considered a promising approach to treating AD based on the relationships among the microbiota, gut, and brain.^[17]^ Ferulic acid (FA) produced by probiotics has antioxidant and anti-inflammatory effects. It can inhibit the formation, deposition, and maturation of amyloid-beta (Aβ) in a dose-dependent manner and delay the progression of AD.[^18^,^19^] Some probiotics (*Lactobacillus plantarum* WCFS1, *Escherichia coli* Nissle, and *Bifidobacterium infantis*) have been documented to enhance the intestinal barrier function by enhancing the tight junction of intestinal epithelial cells, making it difficult for β-amyloid peptide (Aβ), endotoxin, and other substances in the gastrointestinal biological environment to activate the peripheral immune response, and delaying the onset of AD pathological features such as amyloid plaques and neuroinflammation.[^20^,^21^] Accordingly, studying the relationship between AD and intestinal flora can overcome the shortcomings or even complement many candidate AD drugs to provide an optimal treatment option for AD.

To the best of our knowledge, no systematic review or meta-analysis has reported intestinal flora changes at different AD stages. We sought to explore the relationship between intestinal microflora and preclinical stage, mild cognitive impairment and dementia, and identify potential disease prevention or treatment targets.

## 2 Methods and analysis

The study was registered with the International Platform of Registered Systematic Review and Meta-Analysis (INPLASY, registration number INPLASY2021100093). We designed this systematic review and meta-analysis according to the preferred reporting items of the systematic review and meta-analysis program statement.[^22^,^23^]

### 2.1 Qualifying criteria

The inclusion and exclusion criteria in this study were defined based on the population-intervention-comparison-outcome and study design (PICOS) criteria.

### 2.2 Inclusion criteria

#### 2.2.1 Types of studies

Studies on the correlation between Alzheimer disease and intestinal flora were included in the systematic review. The included literature was not limited to the language type, blinding method, or allocation concealment requirements. As long as the included studies are approved by the local institution, we will include this study in the scope of research, including clinical studies and case-control studies.

#### 2.2.2 Subjects

Patients with Alzheimer disease were diagnosed according to NIA-AA (2011) criteria.[^24^,^25^,^26^] According to the typical clinical symptoms of AD, AD patients were divided into 3 groups according to the diagnostic criteria: preclinical stage, mild cognitive impairment, and dementia stage to characterize changes in intestinal flora in the 3 stages of AD (Table 1).

**Table 1:** NIA-AA diagnostic criteria (2011).

Syndromal cognitive stage			
	Cognitively unimpaired	MCI	Dementia
Biomarker profile			
A^-^T^-^(N)^-^	Normal AD biomarkers, cognitively unimpaired	Normal AD biomarkers with MCI	Normal AD biomarkers with dementia
A^+^T^-^(N)^-^	Preclinical Alzheimer pathological change	Alzheimer pathological change with MCI	Alzheimer pathological change with dementia
A^+^T^-^(N)^+^	Alzheimer and concomitant suspected non-Alzheimer pathological change, cognitively unimpaired	Alzheimer and concomitant suspected non-Alzheimer pathological change with MCI	Alzheimer and concomitant suspected non-Alzheimer pathological change with dementia
A^+^T^+^(N)^−^ A^+^T^+^(N)^+^	Preclinical Alzheimer disease	Alzheimer disease with MCI (Prodromal AD)	Alzheimer disease with dementia

Non-Alzheimer continuum profiles are not included in this table^[27]^ because the risk associated with different combinations of T^+^(N)^–^, T^+^(N)^+^, T^–^(N)^+^ among A- individuals has not been established.

#### 2.2.3 Intervention measures

The experimental group consisted of patients in the preclinical stage, mild cognitive impairment, and dementia stage of Alzheimer disease. Acetylcholinesterase inhibitors^[28]^ and NMDA receptor antagonists[^29^,^30^] were the first-line drugs for AD in the experimental group. The control group consisted of healthy subjects without Alzheimer disease. The batches, doses, administration times, and course of treatment were not limited in each group. To eliminate the confounding effect of drugs, the intestinal flora in AD patients was compared before and after treatment.

#### 2.2.4 Outcomes

Included studies on the changes in the gut microbiota of AD patients provided data on the fecal microbiota profile, composition of gut microbiota, changes in fecal fungal or bacterial microbiota, the abundance of opportunistic pathogens, the abundance of beneficial symbiotic bacteria, and diversity of gut microbiota.

### 2.3 Exclusion criteria

AD patients with gastrointestinal diseases such as cancer or other major symptoms such as nausea. Studies where the full-text version was not available. Studies that did not provide clear efficacy evaluation criteria. Studies with no clear dosing or dosage form provided. Comments, brief investigations, case reports, and letters to the editor.

### 2.4 Information source and retrieval strategy

**Figure 1:**
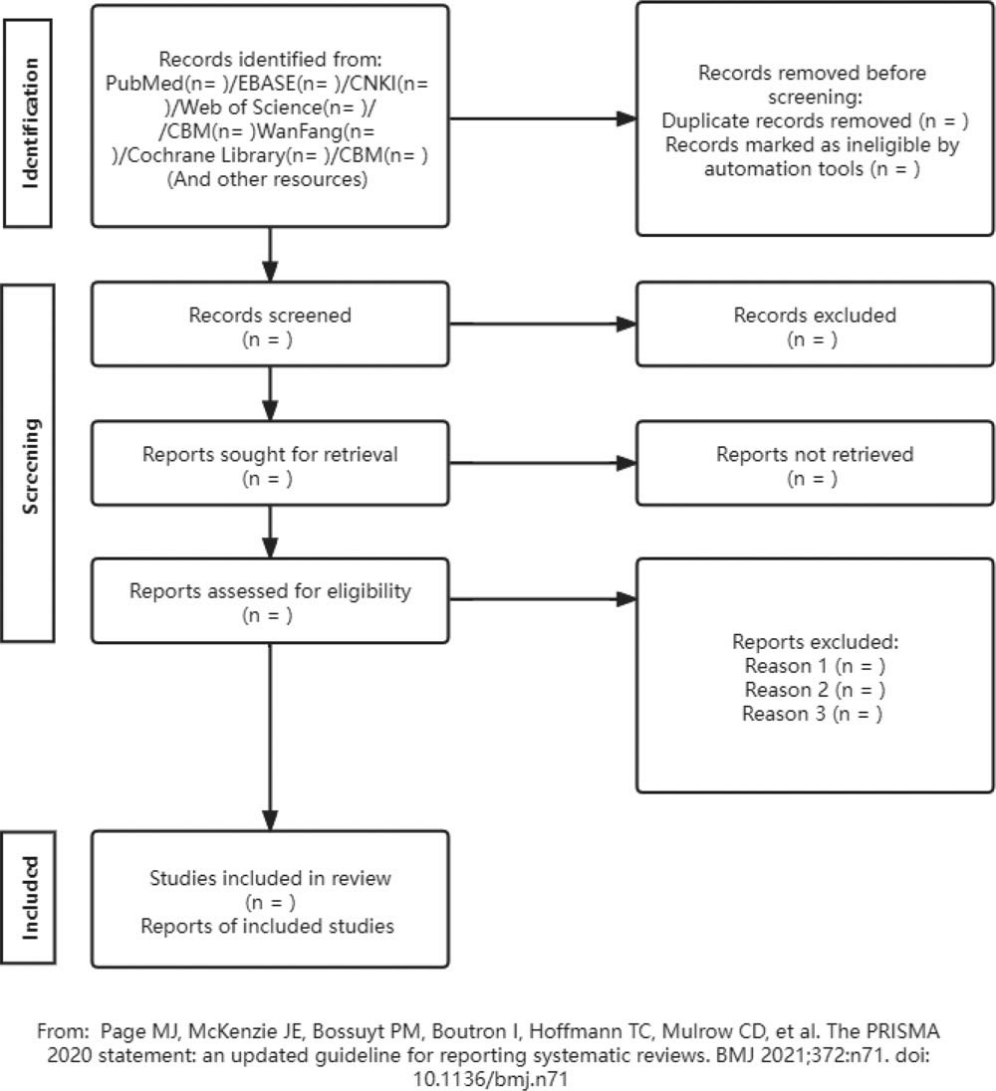
Process of the systematic review.

The search strategy was conducted by CXS and WHR, and points of disagreement were resolved by a discussion with a third reviewer (GMZ). Databases, including PubMed, EBSCO, CNKI, web of science, WanFang, Cochrane Library, CBM, were searched. Databases were searched from inception to January 10, 2022. The following search terms were used: “Alzheimer Disease,” “Preclinical AD,” “Cognitive Dysfunction,” “Dementia,” “Gastrointestinal Microbiome,” etc. For repetitive studies, the complete research report was selected. In cases where a complete report could not be obtained, or the data were incomplete, we contacted the corresponding author to ensure the comprehensiveness of the preliminary search work and prevent the loss of valuable research data. The search strategy of this study is shown in Table 2 (taking the PubMed database as an example). (Fig. 1).

**Table 2:** Search strategy used for PubMed database.

Number	Search terms
1	Search“Alzheimer Disease”[Mesh]
2	((((((((((Alzheimer Dementia[Title/Abstract]) OR (Alzheimer Dementias[Title/Abstract])) OR (Dementia, Alzheimer[Title/Abstract])) OR (Dementia, Senile[Title/Abstract])) OR (Senile Dementia[Title/Abstract])) OR (Dementia, Alzheimer Type[Title/Abstract])) OR (Alzheimer Type Dementia[Title/Abstract])) OR (Alzheimer-Type Dementia (ATD)[Title/Abstract])) OR (Alzheimer Type Dementia (ATD)[Title/Abstract])) OR (Dementia, Alzheimer-Type (ATD)[Title/Abstract]))
3	#1 OR #2
4	Search “preclinical AD”
5	Search“Cognitive Dysfunction”[Mesh]
6	((((((((Cognitive Dysfunctions[Title/Abstract]) OR (Dysfunction, Cognitive[Title/Abstract])) OR (Dysfunctions, Cognitive[Title/Abstract])) OR (Cognitive Impairments[Title/Abstract])) OR (Cognitive Declines[Title/Abstract])) OR (Decline, Cognitive[Title/Abstract])) OR (Declines, Cognitive[Title/Abstract])) OR (Mental Deterioration[Title/Abstract]))
7	#5 OR #6
8	Search“Dementia”[Mesh]
9	((((((((Dementias[Title/Abstract]) OR (Amentia[Title/Abstract])) OR (Amentias[Title/Abstract])) OR (Senile Paranoid Dementia[Title/Abstract])) OR (Dementias, Senile Paranoid[Title/Abstract])) OR (Paranoid Dementia, Senile[Title/Abstract])) OR (Familial Dementia[Title/Abstract])) OR (Dementia, Familial[Title/Abstract]))
10	#8 OR #9
11	#3 OR #4 OR #7 OR #10
12	Search“Gastrointestinal Microbiome”[Mesh]
13	((((((((Gastrointestinal Microbiomes[Title/Abstract]) OR (Microbiome, Gastrointestinal[Title/Abstract])) OR (Gut Microbiome[Title/Abstract])) OR (Gut Microbiomes[Title/Abstract])) OR (Microbiome, Gut[Title/Abstract])) OR (Gut Microflora[Title/Abstract])) OR (Microbiota, Intestinal[Title/Abstract]))
14	#12 OR #13
15	Search: “Randomized Controlled Trial” [Publication Type]
16	CCT,CCS
17	#15 OR #16
18	#11 AND #14 AND #17

### 2.5 Literature screening and data extraction

Based on the above retrieval strategy, 2 researchers (CXS and WHR) independently extracted the data from the included studies. Any points of disagreement were resolved by a discussion with a third reviewer (GMZ). The retrieved articles were imported into Endnote software (Philadelphia, PA) to delete duplicate studies, integrate the literature retrieval results of different databases, establish an information database and download the full texts. If necessary, the author of the original study was contacted by email and telephone to obtain very important information for this study. Then the data were extracted using a predefined data extraction form (such as Microsoft Excel), cross-checked and reviewed, and the reasons for each excluded study were recorded for preliminary screening. Finally, third-party researchers were invited to discuss and study, and opinions were put forward to make the final decision.[^31^,^32^] Data extraction included: the basic information of the included literature (research topic, published journal, year, first author). The basic characteristics and intervention measures of the research object. Key elements of bias risk assessment. Outcome indicators and outcome measurement data of research attention.

### 2.6 Quality and bias assessment

The research methodology, which included the quality and bias risk assessment, was independently assessed by 2 researchers (CXS and WHR) using the Cochrane Risk of Bias tool. If the results were different, third-party researchers (GMZ) were invited to discuss and analyze the source of the bias. According to the quality assessment criteria of Cochrane Handbook for Systematic Reviews, RevMan (Cochrane, London, UK) was used to evaluate the integrity of the methods, whether the random method was correct, whether the distribution of concealment, whether it was used for analysis, and whether the results were complete. Among them, randomized controlled trial studies used the appropriate standard of Cochrane risk bias assessment tool to divide the studies into low risk, high risk, and unknown risk[^33^,^34^] and recorded the basis for judgment.

### 2.7 Statistical analysis

#### 2.7.1 Assessment of heterogeneity

The choice of whether to conduct a meta-analysis and which model to use (fixed or random effects) will depend on the level of statistical heterogeneity assessed by the *I* ^2^ index. A fixed-effects model was used for meta-analysis in the absence of significant heterogeneity (*P* ≥ .1, *I* ^2^ ≤ 0.5). If significant heterogeneity (*P* < .1, *I* ^2^ > 0.5) was present, the source of heterogeneity was first analyzed to exclude the effects of clinical or methodological heterogeneity, and a meta-analysis was performed using a random-effects model. When the meta-analysis could not analyze the data provided by clinical trials, a descriptive analysis was performed.^[35]^ If high heterogeneity was present, sensitivity analysis or subgroup analysis was conducted.

#### 2.7.2 Data synthesis and meta-analysis

This study aimed to study intestinal flora changes in different stages of Alzheimer disease. Accordingly, Alzheimer disease was divided into early, middle, and late stages. To eliminate the effects of drugs on the intestinal flora, we analyzed the clinical manifestations of the 3 stages before and after treatment to improve our understanding of the intestinal flora changes more clearly, and identify probiotics or other factors that can improve the clinical manifestations of AD. The early, middle, and late stages of Alzheimer disease were used as subgroups and the intestinal flora before and after treatment as statistical effects. If there are enough data and outcome indicators in the included study to calculate the comprehensive effect, RevMan software was used for meta-analysis. If high heterogeneity was present in the study, a systematic review was performed to summarize the evidence related to intestinal flora changes during the early, middle, and late stages of Alzheimer disease.

#### 2.7.3 Subgroup and sensitivity analyses

The abundance of intestinal flora in the 3 groups was measured before drug treatment, and the average abundance of intestinal flora in each group was compared with that in the normal population. After treatment, the abundance of intestinal flora in each group was measured again, and the mean value was compared with the mean abundance of flora before treatment and normal flora. Criteria for early, middle, and late AD are based on diagnostic criteria, interventions, and outcomes. If substantial heterogeneity was detected, subgroup analysis and meta-regression analysis were performed to find potential causes. For each excluded study, meta-analysis was conducted again, and the results were compared with those before exclusion. If no significant change was observed during the comparative analysis, the results were stable, otherwise, the results were unstable.

#### 2.7.4 Report deviation assessment

According to Cochrane Handbook, if analysis of >10 studies was conducted, RevMan was used to analyze potential publication bias and generate a funnel plot. If the shape of the plot was a symmetrical inverted funnel, it indicated a small possibility of publication bias. If the funnel plot was asymmetric or incomplete, it indicated that the possibility of publication bias was large.^[36]^

### 2.8 Ethics and dissemination

This study did not require ethical approval since it is a systematic review. The research results will be disseminated by publishing manuscripts in peer-reviewed journals and conducting domestic and international reports.

## 3 Discussion

AD is a neurodegenerative disease with mounting prevalence worldwide during the aging world population. The cognitive dysfunction resulting from AD is a source of burden to patients and families and has a substantial impact on society. At present, the relationship between different stages of Alzheimer disease and intestinal flora remains poorly studied. Most studies only reported the relationship between a single stage of Alzheimer disease and intestinal flora, with no meta-analysis and systematic reviews published. The purpose of this study was to use an optimized methodology to evaluate the relationship between the 3 stages of AD (preclinical stage, mild cognitive impairment stage, and dementia stage) and the heterogeneity in the intestinal flora. Importantly, we analyzed the composition of the intestinal flora, the abundance of opportunistic pathogens, and the abundance of beneficial symbiotic bacteria. By incorporating the intestinal flora changes before and after treatment, probiotics or other reasons closely related to treatment efficacy were screened. According to the exponential effect, the indicators of intestinal bacteria in different AD stages were sorted, and compelling evidence on the relationship between AD stages and intestinal flora was obtained. Our study retrospectively studied the relationship between intestinal microflora and different stages of AD, explained the different effects of different stages of AD on intestinal microflora and vice versa. Our study provides new insights for altering the intestinal flora in AD patients as a therapeutic approach.

## Acknowledgments

The authors thank the editor and anonymous reviewers for their contributions to improving the quality of this article.

## Author contributions

**Conceptualization:** Lijin Ji.

**Data curation:** Chenhao Bi, Xunshu Cheng, Haorui Wang.

**Formal analysis:** Xunshu Cheng, Ke Feng, Lijin Ji, Haorui Wang, Mingzhou Gao.

**Funding acquisition:** Lijin Ji, Mingzhou Gao.

**Investigation:** Zhihuang Zheng, Ke Chen.

**Methodology:** Chenhao Bi, Lijin Ji.

**Project administration:** Lijin Ji.

**Resources:** Mingzhou Gao, Lijin Ji.

**Software:** Mingzhou Gao.

**Supervision:** Saixue Tang, Mingzhou Gao.

**Validation:** Yuanyuan Liu, Mingzhou Gao.

**Writing – original draft:** Xunshu Cheng, Haorui Wang.

**Writing – review & editing:** Xunshu Cheng, Mingzhou Gao.
